# D1 Dopamine Receptor Signaling Is Modulated by the R7 RGS Protein EAT-16 and the R7 Binding Protein RSBP-1 in *Caenoerhabditis elegans* Motor Neurons

**DOI:** 10.1371/journal.pone.0037831

**Published:** 2012-05-21

**Authors:** Khursheed A. Wani, Mary Catanese, Robyn Normantowicz, Muriel Herd, Kathryn N. Maher, Daniel L. Chase

**Affiliations:** 1 Department of Biochemistry and Molecular Biology, University of Massachusetts, Amherst, Massachusetts, United States of America; 2 Molecular and Cellular Biology Program, University of Massachusetts, Amherst, Massachusetts, United States of America; Brown University, United States of America

## Abstract

Dopamine signaling modulates voluntary movement and reward-driven behaviors by acting through G protein-coupled receptors in striatal neurons, and defects in dopamine signaling underlie Parkinson's disease and drug addiction. Despite the importance of understanding how dopamine modifies the activity of striatal neurons to control basal ganglia output, the molecular mechanisms that control dopamine signaling remain largely unclear. Dopamine signaling also controls locomotion behavior in *Caenorhabditis elegans*. To better understand how dopamine acts in the brain we performed a large-scale dsRNA interference screen in *C. elegans* for genes required for endogenous dopamine signaling and identified six genes (*eat-16, rsbp-1, unc-43, flp-1, grk-1, and cat-1*) required for dopamine-mediated behavior. We then used a combination of mutant analysis and cell-specific transgenic rescue experiments to investigate the functional interaction between the proteins encoded by two of these genes, *eat-16* and *rsbp-1*, within single cell types and to examine their role in the modulation of dopamine receptor signaling. We found that EAT-16 and RSBP-1 act together to modulate dopamine signaling and that while they are coexpressed with both D1-like and D2-like dopamine receptors, they do not modulate D2 receptor signaling. Instead, EAT-16 and RSBP-1 act together to selectively inhibit D1 dopamine receptor signaling in cholinergic motor neurons to modulate locomotion behavior.

## Introduction

Dopamine (DA) modulates neural activity by acting through two classes of G protein-coupled receptors. These receptors are expressed in many regions of the brain including the prefrontal cortex where they can affect short-term working memory and in the basal ganglia where they affect motor and reward behaviors [1]–[4]. Defects in DA signaling contribute to neurological disorders that include schizophrenia and Parkinson's disease.

There are five DA receptors in mammals and they are grouped into the D1-like class (D1 and D5 receptors) and the D2-like class (D2, D3, and D4 receptors) based on biochemistry, pharmacology, and amino acid sequence similarity [5]. Biochemically, D1-like receptors can enhance adenylyl cyclase (AC) activity by acting through the G protein subunits Gαs and/or Gαolf and D2-like receptors either do not modulate AC or they inhibit its activity by acting through Gαi/o subunits [6]–[11]. Coupling of DA receptors to specific G protein subunits however is not strict as each receptor can act through several different α subunits depending on the cell type in which the receptor is expressed and α subunit availability [12]–[14].

Binding of DA to its receptor induces the coupled G protein α subunit to exchange GDP for GTP, causing the separation of the α subunit from the βγ complex. Both freed α and βγ subunits can modulate the activity of downstream molecules. The best studied of the α subunit targets is AC which, when activated, converts ATP to cAMP to directly modulate the activity of protein kinase A (PKA). PKA then phosphorylates a number of target molecules including AMPA and NMDA receptors to affect their activity and/or localization. The βγ complex is also capable of acting on downstream targets including phospholipase C (PLC) and ion channels [15]. While DA receptors often couple to Gαs or Gαi/o to modulate AC activity, there is evidence that the D5 receptor and the D1/D2 heterodimer can act through Gαq to activate PLCβ and generate IP_3_ and diacylglycerol (DAG) [16], [17]. The physiological targets of signaling downstream of D1-like receptors and Gαq are currently only partly described but may include the activation of calcium/calmodulin protein kinase II (CamKII) [18].

Signaling activated by DA receptors continues until the GTP bound to the α subunit is hydrolyzed. To rapidly shut down G protein receptor signaling the weak intrinsic GTPase activity of the α subunit is enhanced by a family of regulators of G protein signaling proteins (RGS proteins). RGS proteins bind the α subunit and stabilize the transition state of hydrolysis speeding up the rate of GTP hydrolysis more than 40-fold [19], [20]. While RGS proteins play a critical role in G protein signaling it is not clear precisely how they are regulated.

DA also modulates neural activity to affect locomotion in *C. elegans.* The mammalian enzymes involved in DA synthesis, vesicle loading and reuptake by neurons all have *C. elegans* homologs, and mutants for each of these homologs have previously been analyzed [21], [22]. DA is synthesized in just eight of the 302 neurons found in *C. elegans* and these eight neurons appear to be mechanosensory [23], [24]. They release DA when the animal encounters a food source such as bacteria [24]. DA released from these neurons binds to D1-like (DOP-1) and D2-like (DOP-3) receptors (similar in sequence to those found in the mammalian brain) expressed in the motor neurons to modulate locomotion behavior. DA inhibits locomotion behavior by acting through the DOP-3 receptor but can also enhance locomotion by acting through the DOP-1 receptor [25]. We have shown previously that the DOP-3 receptor couples to the G protein α subunit GOÃ1 (80% identical to the mammalian Gαo) and the DOP-1 receptor couples to the α subunit EGL-30 (80% identical to the mammalian Gαq) but few other downstream targets have been identified [25].

Increased concentrations of synaptic DA, caused either by the application of exogenous DA [25] or by mutations of the DA transporter *dat-1*, [26], cause animals to become paralyzed. Regardless of the source of DA, paralysis is caused, at least in part, by hyperactivation of the DOP-3 receptor expressed in the motor neurons [26], [27]. To begin to better understand how DA modulates neural activity in the brain we performed a large-scale dsRNAi screen in *C. elegans* searching for genes that were required for endogenous DA signaling. We identified six genes from this screen that encode UNC-43 (the homolog of mammalian calcium/calmodulin-dependent protein kinase CamKII), CAT-1 (homolog of the mammalian monoamine transporter VMAT2), GRK-1 (homolog of mammalian G protein receptor kinase 4 family), FLP-1 (an FMRF-amide related peptide), EAT-16 (the homolog of mammalian R7 RGS protein RGS9), and RSBP-1 (homolog of the mammalian R7 RGS binding protein R7BP). Here we have characterized the function of EAT-16 and RSBP-1 and show that they are both necessary for endogenous DA signaling. Using a combination of genetic and behavioral studies that allowed us to examine the physiological roles of EAT-16 and RSBP-1 in single cell types, we found that EAT-16 and RSBP-1 function together in cholinergic motor neurons to modulate D1-like (DOP-1) receptor signaling *in vivo*.

## Results

### Large-scale dsRNA interference screen identifies genes required for endogenous DA signaling

We performed a large-scale dsRNA interference (dsRNAi) screen to identify new genes required for DA signaling. For this screen we used *dat-1* mutant animals. *dat-1* encodes a DA transporter similar to that found in mammals which is capable of transporting excess DA from the synapse back into dopaminergic cells [28]. Mutations in *dat-1* result in increased synaptic DA levels and caused an abnormal locomotion behavior known as swimming-induced paralysis or SWIP [26]. Wild-type animals when placed in water swim continuously for more than 30 minutes while *dat-1* mutants become paralyzed within 6–10 minutes of swimming [26]. The reduced rate of locomotion observed in *dat-1* mutants is caused by excess DA acting through the D2-like DOP-3 receptor in motor neurons that innervate body muscle cells [26], [27]. We fed *dat-1* mutant animals dsRNA targeted against *C. elegans* genes and identified those genes whose expression was required for *dat-1* mutants to exhibit the SWIP behavior (thus a SWIP suppressor screen). In this screen we expected to identify genes that were either required for DA synthesis and release from dopaminergic neurons or that were required for modulating DA signaling in dopamine-receptive neurons. Because *C. elegans* neurons are refractory to RNA-mediated interference, we combined the *dat-1* mutation with mutations in two genes that enhance RNAi effects in neurons but that do not affect SWIP behavior [29], [30] to generate the strain XP292 (genotype: *dat-1 (ok157); eri-1 (mg366); lin-15 (n744*)).

**Figure 1:**
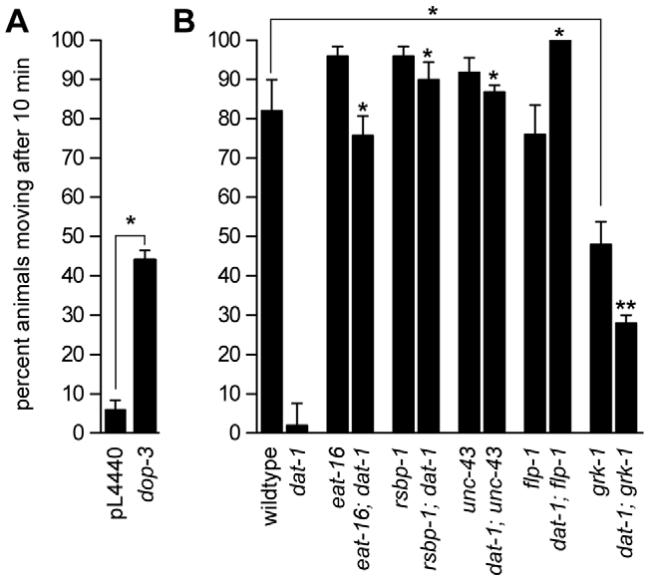
Quantitative analysis of SWIP behavior in knockdown or null mutants of dopamine signaling genes. (A), SWIP behavior of XP292 animals fed dsRNA-expressing bacteria. The dsRNA fed to XP292 animals is indicated below each bar. XP292 animals fed bacteria containing empty vector pL4440 become paralyzed within 10 min of swimming while animals fed bacteria expressing dsRNA that targets *dop-3* continue to swim under these conditions. Students t test, asterisks indicate p<0.001. (B), SWIP behavior of animals with null mutations in genes identified in the dsRNAi screen. EAT-16, RSBP-1, UNC-43, FLP-1, AND GRK-1 are required for SWIP behavior caused by *dat-1* mutation. Each measurement shown in either panel represents the mean of five trials of 10 L4 animals each for a total of 50 animals per dsRNA fed or mutant strain. Error bars represent standard error of the mean. All strains were compared using one-way ANOVA with Bonferroni's post hoc test. Single asterisks indicate p<0.001. Double asterisks indicate p<0.01. Except where indicated by the connecting line, all statistical comparisons shown are to *dat-1* single mutants. We note that *grk-1* single mutants showed significant SWIP when compared to wild-type animals but that *grk-1* also suppressed *dat-1*-induced SWIP.

In the screen we placed 30±10 L1 stage XP292 larvae on agar plates containing bacteria that expressed a single dsRNA and allowed them to feed and reproduce for six days to generate first generation (F1) broods of ≥500 animals. When the oldest F1 animals were L4 stage larvae we washed the entire population of animals off the food plates and assayed them for SWIP behavior. In initial trials with small populations of animals only 6%±2% of XP292 animals fed bacteria containing empty vector (pL4440) were moving after 10 minutes while 44%±2% of *dop-3* fed animals were capable of movement after this time period (Figure 1A). In the screen we selected as positive “hits” any gene that suppressed SWIP behavior such that >40% of animals were moving after 10 minutes.

We have so far surveyed 19% of all *C. elegans* genes (3,610 total genes). Of these, dsRNAi of 681 genes (∼19% of genes tested) caused a lethal phenotype, which we define as the inability of dsRNA-fed animals to sustain a brood. The three most common terminal lethal phenotypes observed included: 1) larval arrest; 2) failure of animals to produce eggs; and 3) the production of eggs that failed to hatch. We also identified six genes required for the SWIP phenotype (Table 1).

**Table 1:** Genes identified in the dsRNAi screen.

Gene	Protein	Human homolog
*unc-43*	calcium/calmodulin-dependent protein kinase	CamKII
*grk-1*	G protein receptor kinase	GRK4-6
*flp-1*	FMRFamide related peptide	none*
*eat-16*	R7 RGS protein	RGS6, 7, 9, 11**
*rsbp-1*	R7 RGS binding protein	R7BP
*cat-1*	monoamine vesicular transporter	VMAT2

* FLP-1 encodes up to eight invertebrate-specific FMRFamide-related peptides.

** EAT-16 is similar in both sequence and domain structure to all four human R7 RGS protein family members but is not clearly more related to one member than the others.

XP292 animals fed dsRNA targeting all six identified genes (*eat-16*, *rsbp-1, unc-43*, *flp-1*, *grk-1, and cat-1*) resulted in >50% suppression of the *dat-1* SWIP phenotype during both the initial screen and in subsequent retests. *cat-1* encodes the monoamine vesicle transporter and is required to load DA into synaptic vesicles [31]. We expected to identify genes involved in the synthesis, vesicle loading and release of DA and so the identification of *cat-1* indicated that the screen could identify genes required for DA signaling. We selected one mutant allele to represent each of the five remaining genes, combined these null mutations with the *dat-1* mutation, and tested the resulting double mutants for SWIP behavior to confirm our screen results (Figure 1B). The swimming behavior of *eat-16*, *rsbp-1*, *unc-43*, and *flp-1* single mutants was similar to each other and to wild-type animals while *grk-1* showed some defects in swimming. Regardless, we found that mutations in each of the five genes identified in the dsRNA screen suppressed the SWIP phenotype of *dat-1* single mutant animals (*dat-1* single mutants 2%±2% moving, *eat-16; dat-1* double mutants 76%±5%, *rsbp-1; dat-1* double mutants 90%±4%, *dat-1; unc-43* double mutants 87%±2%, *dat-1; flp-1* double mutants 100%±0%, *dat-1; grk-1* double mutants 28%±2%) (Figure 1B). These results indicate that RSBP-1, EAT-16, UNC-43, FLP-1, and GRK-1 are each required for the SWIP behavior caused by excess endogenous DA signaling in *dat-1* mutants and suggest that these proteins mediate endogenous DA signaling.

*eat-16* encodes an R7 class RGS protein which acts as a GTPase accelerating protein for Gα subunits and is similar to members of the mammalian R7 RGS9 protein family [32]. *rsbp-1* encodes an R7 anchoring protein homologous to mammalian R7BP [33]. Our previous analysis of DA signaling implicated EAT-16 as a regulator of D1 (DOP-1) signaling [25] and, while no particular signaling pathway was implicated, others have demonstrated that EAT-16 and RSBP-1 act together to control aspects of locomotion and egg-laying behavior in *C. elegans* [33]. The interaction between EAT-16 and RSBP-1 is similar to that described for RGS9-2 and R7BP in mammals [34] where it has been suggested that the RGS9-2/R7BP complex regulates D2 receptor signaling [35]. Thus we decided to investigate whether RSBP-1 acts together with EAT-16 to modulate DA signaling in *C. elegans*, and if so, whether it regulates D1 (DOP-1) or D2 (DOP-3) signaling.

### *rsbp-1* mutants are defective in other DA-mediated behaviors

RSBP-1 is the sole *C. elegans* homolog of the mammalian R7 RGS binding proteins R7BP and R9AP. In mammals R9AP is expressed exclusively in the retina where it anchors the R7 RGS protein RGS9-1 to photoreceptor outer segment disk membranes to modulate Gα_t_ activity in response to light [36]. In contrast, R7BP is widely expressed in the brain with higher expression levels in richly dopamine-innervated regions including the striatum and olfactory tubercle, where it is coexpressed and binds to the RGS7 and RGS9-2 proteins [37]–[39]. While R7BP can bind to both RGS7 and RGS9-2 in brain extracts, most R7BP found in the striatum is bound to RGS9-2 and this physical interaction is required for the stability and function of the RGS9-2 protein [40]. The coexpression of R7BP, RGS7, and RGS9-2 in DA-receptive regions of the brain led others to investigate whether these molecules were able to modulate DA signaling [35], [41]–[43]. The results of these studies suggest that RGS9-2, RGS7, and R7BP can all modulate locomotion behavior and an animal's response to addictive drugs, but the cellular and molecular mechanisms of their action on DA signaling remain largely unclear.

**Figure 2:**
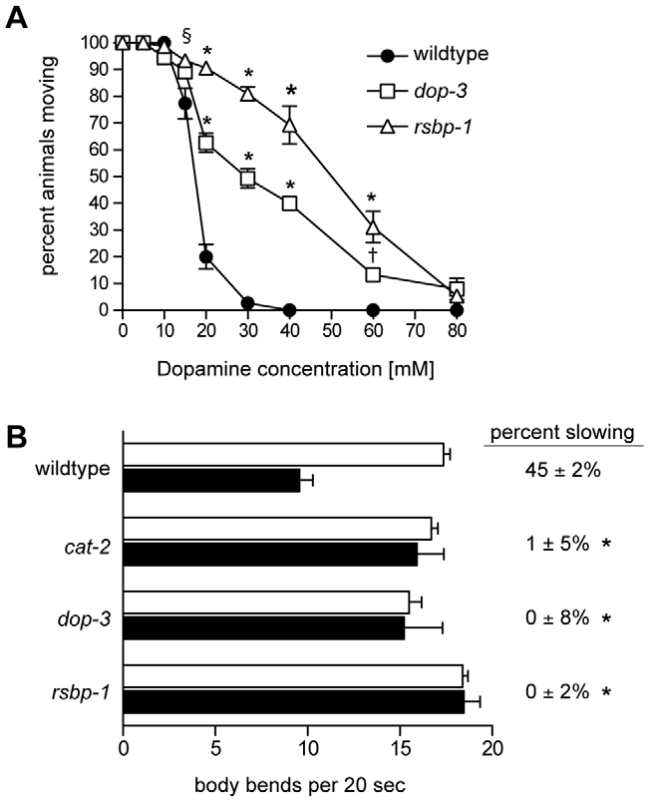
Analysis of dopamine signaling defects in *rsbp-1* mutants. (A), Dose-response curves measuring locomotion behavior in response to exogenous dopamine. Shown are the percentages of animals moving 20 min after being placed on agar plates containing the indicated concentrations of dopamine. Each data point represents the mean ± standard error of the mean for three trials totaling at least 75 animals. (Two way ANOVA with Bonferroni post hoc test, *p<0.0001, ^§^p<0.01, ^†^p<0.05 when compared to the wildtype). (B), Quantitative analysis of basal slowing behavior. For each strain, locomotion rates in the absence of bacteria (white bars) and presence of bacteria (black bars) were calculated as the average of 30 observations. Error margins shown indicate 95% confidence intervals. Asterisks indicate values significantly different from the 45% slowing seen in the wildtype. (One-way ANOVA with Bonferroni post hoc test, asterisks indicate p<0.001). The percent slowing in the presence of bacteria for each strain is shown at the right. *rsbp-1* mutants are defective in endogenous dopamine signaling.

To begin to investigate the role of RSBP-1 in DA signaling we examined the behavioral response of *rsbp-1* mutants to exogenous DA (Figure 2A). Locomotion rate is modulated by DOP-1 and DOP-3 receptors acting in cholinergic and GABAergic motor neurons that innervate body wall muscle cells [25], [27]. Exogenous DA also acts through these receptors to have opposite effects on locomotion: DOP-3 signaling inhibits locomotion and DOP-1 signaling enhances locomotion [25]. Wild-type animals exposed to exogenous DA slow their locomotion rate until, at sufficiently high DA concentrations, they are unable to move and appear paralyzed, suggesting that DOP-3 signaling prevails over DOP-1 signaling in the presence of high concentrations of DA [25], [44] (Figure 2A). Consistent with this, *dop-3* mutants are resistant to exogenous dopamine and *dop-1* mutants are hypersensitive to the effects of exogenous DA on locomotion rate [25](Figure 2A). We found that *rsbp-1* mutants, like *dop-3* mutants, were resistant to the paralytic effects of exogenous dopamine suggesting that RSBP-1 mediates the effects of exogenous DA and thus may (like DOP-3) mediate endogenous DA signaling (Figure 2A).

This result is contrary to what we would expect if RSBP-1 were acting with an R7 RGS protein to inhibit the activity of DOP-3. Indeed, we would expect *rsbp-1* mutations to cause behavioral defects opposite to those caused by mutations in the signaling pathways that they inhibit. Therefore this result suggests that RSBP-1 does not inhibit signaling by the D2-like DOP-3 receptor. Notably, mutations in the D1-like DOP-1 receptor cause behavioral defects that are opposite to those caused by mutations in the DOP-3 receptor [25] and thus opposite to those caused by mutations in RSBP-1 suggesting that RSBP-1 may inhibit D1-like DOP-1 signaling.

To further support a role for RSBP-1 in DA signaling we tested *rsbp-1* mutants for defects in basal slowing response, a behavior that is dependent on endogenous DA signaling [24]. Wild-type animals slow their locomotion rate when they encounter a source of food such as a bacterial lawn and this response, known as basal slowing, requires DA signaling [24](Figure 2B). We found that *rsbp-1* mutants, like *cat-2* mutants (*cat-2* encodes tyrosine hydroxylase, required for DA biosynthesis), and mutants in the DA receptor *dop-3* fail to slow in response to food (Figure 2B) (wild-type animals show 45% slowing ±2%, *cat-2* mutants 1% slowing ±5%, *dop-3* mutants 0% slowing ±8%, *rsbp-1* mutants 0% slowing ±2%). As the ability of animals to slow in response to food is absolutely dependent upon endogenous DA signaling, these results indicate that RSBP-1 is required for dopamine signaling *in vivo* and again suggest that RSBP-1 is not acting as an inhibitor of D2-like DOP-3 receptor signaling as both *rsbp-1* and *dop-3* mutants fail to slow in response to food. Like *rsbp-1* mutants, *eat-16* mutants also fail to slow in response to food [25] suggesting that RSBP-1 and EAT-16 act together to modulate endogenous DA signaling.

### RSBP-1 is expressed in DA-receptive neurons that control locomotion

**Figure 3:**
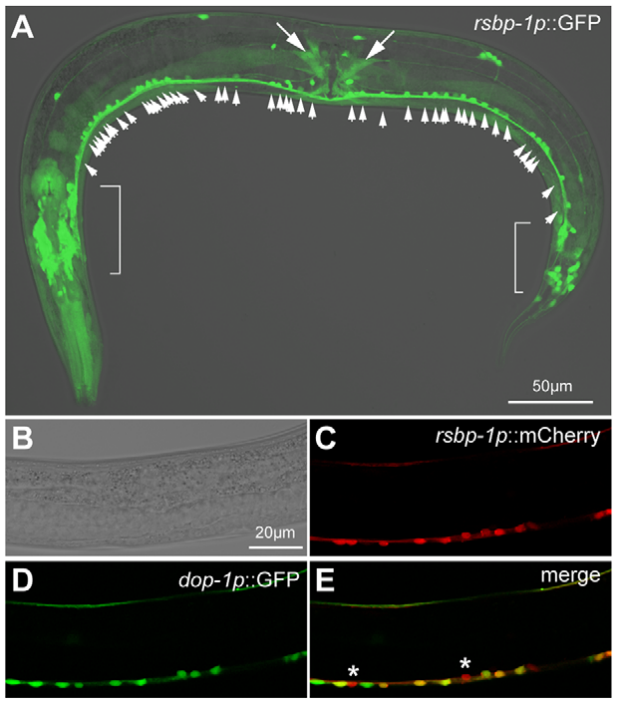
Fluorescence of animals expressing *rsbp-1* and *dop-1* promoter transgenes. (A), young adult transgenic animal showing expression of *rsbp-1p*::GFP in neurons of the head and retrovesicular ganglia (left bracket), pre-anal ganglia and tail neurons (right bracket), and vulval muscle cells (large arrows). Expression is also seen in the cell bodies and processes of ventral cord motor neurons. (small arrows indicate positions of the ventral cord neuron cell bodies). Faint green fluorescence can also be seen in body-wall and pharyngeal muscle cells. (B–D), high-power magnification images of the ventral cord area of a double transgenic animal expressing *rsbp-1p*::mCherry and *dop-1p*::GFP transgenes. In all images dorsal is up and anterior is left. (B), Nomarski image of double transgenic animal shown in panels C–E. (C), red fluorescence of the mCherry protein expressed from the extrachromosomal transgene *rsbp-1p*::mCherry. (D), green fluorescence of GFP protein expressed from the chromosomally integrated transgene *dop-1p*::GFP. (E), merged image showing coexpression of *rspb-1* and *dop-1* transgenes in cholinergic motor neurons. Asterisks indicate the positions of cell bodies of GABAergic motor neurons that express *rsbp-1p*::mCherry but not *dop-1p*::GFP. RSBP is expressed in both cholinergic and GABAergic motor neurons of the ventral cord and is thus coexpressed with both DOP-1 and DOP-3 receptors. Some cells of the ventral cord shown in panel E express the *dop-1p*::GFP but not the *rsbp-1p*::mCherry transgene. The relative position of these non-mCherry-expressing cells varies among transgenic animals suggesting that the lack of expression of the *rsbp-1p*::mCherry transgene in some cells that express the *dop-1p*::GFP transgene is due to random loss of the extrachromosomal transgene during cell division and not due to restricted expression of *rsbp-1*.

In order for RSBP-1 to regulate DA signaling pathways (either D1- or D2-like) it must be expressed in neurons that also express the DA receptors. Thus we examined the expression pattern of RSBP-1. The expression patterns of the DOP-1 and DOP-3 receptors have been already examined [25], [45]. We generated transgenes in which the promoter for *rsbp-1* was used to direct the expression of the green and red fluorescent proteins GFP and mCherry, respectively (*rsbp-1p*::GFP and *rsbp-1p*::mCherry transgenes). These reporter transgenes were separately injected into animals and transgenic progeny were inspected for fluorescence (Figure 3). Similar to a previous analysis of *rsbp-1* expression [33], we found that the reporter transgene was expressed in head and tail neurons and motor neurons of the ventral cord that innervate body-wall muscle cells (Figure 3A). We also observed expression in vulval, pharyngeal, and body-wall muscle cells (Figure 3A). We previously showed that DA modulates locomotion behavior by acting through the DOP-3 receptor, which is expressed in both GABAergic and cholinergic motor neurons of the ventral cord and by acting through the DOP-1 receptor, which is expressed in the cholinergic motor neurons but not the GABAergic motor neurons [25]. The *rsbp-1p*::GFP transgene was expressed in many ventral cord motor neurons (Figure 3A). Only cholinergic and GABAergic motor neuron cell bodies are found in the ventral cord of *C. elegans*. There are 44 cholinergic motor neurons and 13 GABAergic motor neurons located in the ventral cord between the retrovesicular ganglia and pre-anal ganglia of *C. elegans* [46]. We consistently found *rsbp-1p*::GFP expression in >45 ventral cord neurons between these ganglia indicating that RSBP-1 was expressed in both cholinergic and GABAergic motor neurons (Figure 3A). We verified the expression of the *rsbp-1p* reporter transgenes in both GABAergic and cholinergic motor neurons in two ways. First, we generated *rsbp-1p*::GFP, *unc-47p*::mCherry double transgenic animals and examined these animals for coexpression of GFP and mCherry in ventral cord motor neurons. The *unc-47p*:: mCherry transgene is expressed only in GABAergic cells (*unc-47* encodes the transporter required for loading GABA into synaptic vesicles; [47]). We found that *rsbp-1p*::GFP was coexpressed in the ventral cord neurons with this GABAergic-specific marker (data not shown). Second, we generated *rsbp-1p*::mCherry, *dop-1p*::GFP double transgenic animals and examined these animals for coexpression of GFP and mCherry in ventral cord motor neurons. *dop-1p::*GFP is expressed in the cholinergic, but not the GABAergic neurons, of the ventral cord [25] and we found that the *rsbp-1p::*mCherry reporter transgene was coexpressed with *dop-1p::*GFP in the cholinergic ventral cord neurons (Figure 3B–E). In these double transgenic animals *dop-1p*::GFP transgene is stably integrated into the chromosome and therefore this transgene is present in all cells of the animal and caused GFP expression in all cholinergic motor neurons. In contrast, the *rsbp-1p*::mCherry transgene is present as an extrachromosomal array. Extrachromosomal arrays are not stable and can be lost as the result of unequal partitioning of the array between daughter cells during development resulting in a mosaic expression pattern of the transgene. Thus the lack of mCherry expression in individual motor neurons could be due to loss of the transgene and is not necessarily an indication of a lack of *rsbp-1* promoter activity. However, since *dop-1p::*GFP is active in all cholinergic motor neurons the neuron cell bodies in Figure 3E that express mCherry but not GFP must be GABA neurons. The results presented here indicate that RSBP-1 is expressed in GABAergic motor neurons (with the DOP-3 receptor) and is also expressed in cholinergic motor neurons (with both DOP-1 and DOP-3 receptors).

### RSBP-1 functions in the cholinergic motor neurons to mediate dopamine signaling

**Figure 4:**
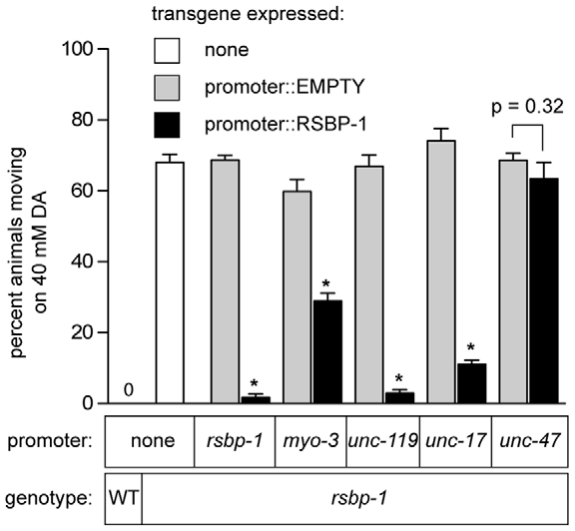
Rescue of *rsbp-1* function by transgenic expression using cell-specific promoters. Animals were tested for paralysis as in Figure 2A, except that a single concentration of DA was used (40 mM). For control, non-transgenic animals (white bars), each measurement shown represents the mean and 95% confidence interval of the mean for at least 75 animals. Gray and black bars represent measurements from *rsbp-1* mutants carrying transgenes. The promoters used for transgene expression are indicated at the bottom. Gray bars represent measurements form control strains carrying empty vector transgenes, which have promoters but no RSBP-1 sequences. We observed no significant differences in the response of these transgenic animals when compared to each other or to *rsbp-1* null mutants that lacked transgenes (p<0.001). Black bars represent measurements from strains carrying transgenes from which the promoters express RSBP-1. For each transgene, measurements of at least 75 animals for each of two or three lines were averaged, and the means and 95% confidence intervals are shown. Asterisks indicate that RSBP-1 expression gave significant rescue compared to control animals that contained promoter but not RSBP-1 coding sequence. Asterisk indicates p<0.001. The intermediate response of *myo-3p*::RSBP-1 animals was different from both *unc-47p*::RSBP-1 animals (p<0.001) and from all other rescue strains (p<0.001). All comparisons done using one-way ANOVA with Bonferroni post hoc test. The *unc-17* promoter gave nearly complete rescue of *rsbp-1* while the *unc-47* promoter had no significant effect (p = 0.32, student's t test) on behavior indicating that RSBP-1 acts in the cholinergic motor neurons and not the GABAergic neurons to mediate DA signaling.

To help determine which DA receptor is modulated by RSBP-1, we next determined where RSBP-1 functions to mediate dopamine signaling. For this we used promoters whose activity is restricted to specific cells of the animal to drive the expression of RSBP-1 and tested the ability of such transgenes to rescue the DA resistance phenotype observed in *rsbp-1* mutants in response to 40mM DA (Figure 4). At 40mM DA, *rsbp-1* mutants were resistant to paralysis (68%±2% moving animals) while wild-type animals were completely paralyzed (0%±0% moving animals). We first expressed RSBP-1 from its own promoter and found that this transgene was capable of complete rescue of DA response (Figure 4). *rsbp-1* null animals carrying a transgene that expressed RSBP-1 coding sequence from its native promoter (transgene designation: *rsbp-1p::*RSBP-1) showed DA sensitivity similar to wild-type animals (2%±1% animals moving) while transgenic animals that carried a transgene that included the *rsbp-1* promoter but which lacked any *rsbp-1* coding sequence (transgene designation: *rsbp-1p*::EMPTY) showed no rescue (69%±1% moving). Because RSBP-1 was expressed in both neurons and muscle cells (Figure 3) and both cell types are required for locomotion behavior, we next expressed RSBP-1 from the muscle-specific promoter *myo-3p*, which is active in all body-wall muscle cells [48]. This transgene (*myo-3p::*RSBP-1) showed some rescue of DA sensitivity (29%±2% animals moving) compared with transgenic animals that expressed the control transgene *myo-3p::*EMPTY (60%±3% animals moving) suggesting that RSBP-1 can function in muscle cells to affect DA response. Next we expressed RSBP-1 from the *unc-119* promoter, which is active in all neurons but is not active in muscle cells [49]. This transgene, (*unc-119p::*RSBP-1), strongly rescued the *rsbp-1* mutant defect such that only 3%±1% of the transgenic animals were able to move compared to 67%±3% for animals that expressed the control transgene (*unc-119p*::EMPTY). Thus, RSBP-1 functions in both neurons and muscle cells to affect locomotion but its primary site of function in DA signaling is in neurons. Because the DOP-1 and DOP-3 receptors are differentially expressed in the cholinergic and GABAergic motor neurons we wanted to test whether *rsbp-1* expression in the cholinergic or the GABAergic ventral cord motor neurons was sufficient to rescue the *rsbp-1* DA signaling defect. For this we used the *unc-17* and *unc-47* promoters, which are active in the cholinergic and GABAergic motor neurons, respectively [47], [50]. Expression of an *unc-17p*::RSBP-1 transgene in the cholinergic motor neurons was sufficient to rescue the DA sensitivity of *rsbp-1* mutants (11%±1% animals moving), compared to 74%±3% for animals that expressed the *unc-17p::*EMPTY transgene. Finally, we found that expression of RSBP-1 in the GABAergic cells using the *unc-47p*::RSBP-1 transgene failed to rescue the DA sensitivity of *rsbp-1* mutants (68%±2% animals moving) compared to animals that expressed the control transgene *unc-47p*::EMPTY (63%±5% animals moving). These data clearly indicate that RSBP-1 functions in the cholinergic and not the GABAergic motor neurons to mediate DA signaling.

The DOP-1 and DOP-3 receptors control locomotion rate in response to exogenous DA, and of these two receptors, DOP-3 is the only receptor expressed in the GABA motor neurons. Therefore our data strongly suggest that RSBP-1 does not modulate D2-like DOP-3 receptor signaling; at least not in GABA motor neurons.

### RSBP-1 acts with the R7 RGS protein EAT-16 and not with EGL-10 to modulate DA signaling

**Figure 5:**
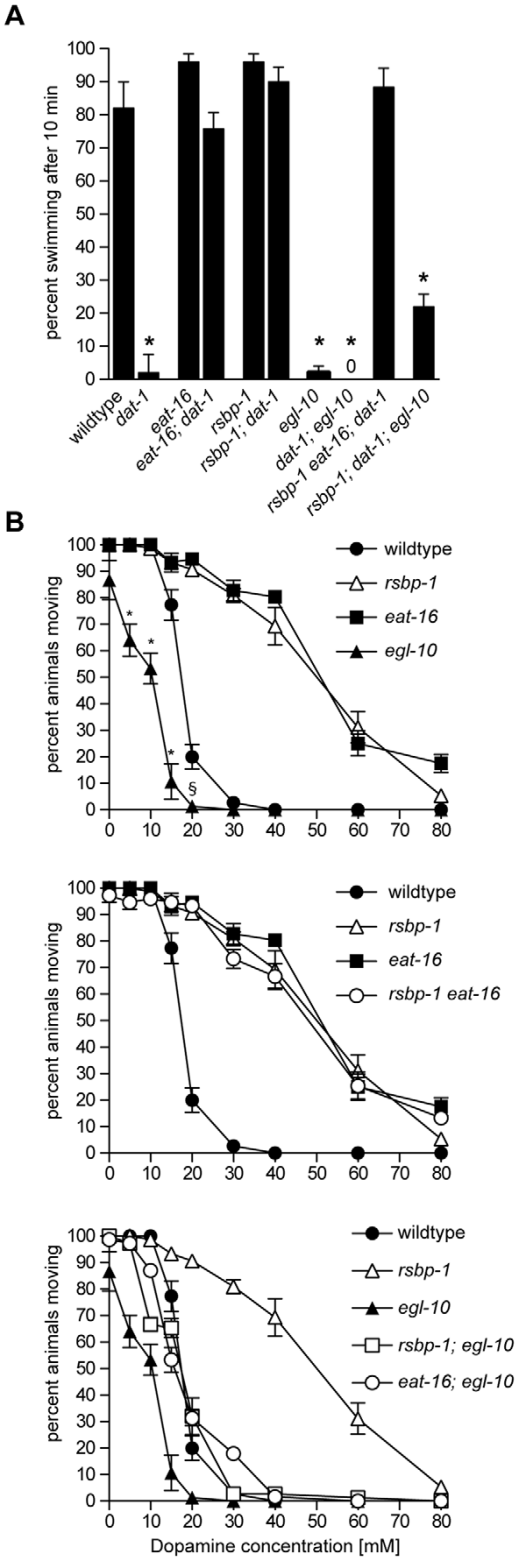
Quantitative behavioral analysis of wild-type and *rsbp-1*, *eat-16*, and *egl-10* mutant animals. (A), SWIP behavior of wild-type or mutant animals. Each measurement shown represents the mean of five trials of 10 L4 animals each for a total of 50 animals per strain. Error bars represent 95% confidence intervals. All strains were compared using one-way ANOVA with Bonferroni post hoc test. Only *dat-1* and *egl-10* single mutants, *dat-1; egl-10* double mutants, and *rsbp-1*; *dat-1*; *egl-10* triple mutants were statistically different from the wildtype (p<0.001). (B), Dose-response curves measuring paralysis induced by exogenous dopamine. Shown are the percentages of animals moving 20 min after being placed on agar plates containing the indicated concentrations of dopamine. Each data point represents the mean ± standard error of the mean (s.e.m.) for three trials totaling at least 75 animals. The responses of *eat-1* and *rsbp-1* mutants are not statistically different from each other at any concentration of dopamine. The response of *egl-10* mutants is significantly different from the wild-type at the indicated concentrations of dopamine (Two way ANOVA with Bonferroni post hoc test, *p<0.0001, ^§^p<0.01).

In mammals the R7BP protein can interact with all four R7 RGS proteins in brain extracts [39]. While this suggests that R7BP-RGS protein complexes are important for modulating G protein receptor signaling in the brain, the physiological significance of these interactions is not yet clear. Since there are two R7 RGS proteins in *C. elegans* (EAT-16 and EGL-10) we sought to determine whether they both acted with RSBP-1 to mediate DA signaling. Thus we tested null mutations in *eat-16* and *egl-10* for defects in DA-specific behaviors and compared them directly to the behavioral defects observed in *rsbp-1* mutants (Figure 5). First we tested animals for defects in SWIP, a behavior controlled by endogenous DA signaling. As shown earlier in Figure 1B, mutation in *eat-16*, like the mutation in *rsbp-1*, suppressed the SWIP phenotype of *dat-1* mutants (Figure 5A). In stark contrast however, we found that *egl-10* mutants failed to suppress the SWIP phenotype of *dat-1* mutants as no *dat-1; egl-10* double mutant animals were moving after 10 minutes of swimming (Figure 5A). That *rsbp-1* and *egl-10* mutations had opposite effects on SWIP behavior suggests that RSBP-1 and EGL-10 do not act together to modulate endogenous DA signaling. We note that mutation of *egl-10* alone caused SWIP. We suggest that this is caused, at least in part, by unregulated DOP-3 activity, consistent with previous reports indicating that: 1) EGL-10 inhibits DOP-3 signaling [25]; and 2) SWIP is caused by excess DA signaling through DOP-3 [26], [27].

To examine the functional interaction between RSBP-1 and the two R7 RGS proteins more directly we tested *rsbp-1 eat-16; dat-1* and *rsbp-1; dat-1; egl-10* triple mutants for SWIP (Figure 5A). We found no significant difference in SWIP behavior of *rsbp-1 eat-16; dat-1* triple mutants compared to either *rsbp-1; dat-1* double mutants or *eat-16; dat-1* double mutants, consistent with *rsbp-1* and *eat-16* acting together to modulate DA signaling. In contrast, *rsbp-1; dat-1; egl-10* triple mutants exhibited a SWIP phenotype that was significantly different from that of *rsbp-1; dat-1* double mutants and was intermediate between that observed for *dat-1* single and *rsbp-1; dat-1* double mutants indicating that *rsbp-1* and *egl-10* mutations have opposite effects on *dat-1*-induced SWIP. Thus, using SWIP behavior as a measure of protein function, it appears that RSBP-1 acts with EAT-16 and not with EGL-10 to modulate endogenous DA signaling.

We then tested *eat-16* and *egl-10* mutants for defects in response to exogenous dopamine. Again, like *rsbp-1* mutants, *eat-16* mutants were resistant to exogenous DA while *egl-10* mutants showed the opposite effect and were more sensitive to exogenous dopamine than wild-type animals (Figure 5B, top panel). Again, the opposite effects of *rsbp-1* and *egl-10* mutations indicate that RSBP-1 and EGL-10 do not act together to modulate DA signaling.

Because mutations in *rsbp-1* and *eat-16* caused similar behavioral defects, we wanted to test whether RSBP-1 and EAT-16 always functioned together to mediate DA signaling. Porter and Koelle [33] previously showed that EAT-16 protein levels are reduced (but importantly were not eliminated) in *rsbp-1* null mutants, but they did not examine RSBP-1 protein levels in *eat-16* null mutants. To determine whether RSBP-1 or the residual EAT-16 present in *rsbp-1* mutants was able to modulate DA signaling, we examined the response of *rsbp-1 eat-16* and *rsbp-1; egl-10* double mutants to exogenous DA (Figure 5B, two lower panels). We found that *rsbp-1 eat-16* double mutants behaved in a manner that was indistinguishable from either *rsbp-1* or *eat-16* single mutants (Figure 5B, middle panel) suggesting that the two proteins only act together to modulate DA signaling. These data also suggest that the residual EAT-16 present in *rsbp-1* null mutant animals does not modulate DA signaling and may be inactive. In contrast, we found that *rsbp-1; egl-10* double mutants showed a sensitivity to DA that was similar to wild-type animals, clearly showing that EGL-10 and RSBP-1 do not act together to modulate DA signaling (Figure 5B, bottom panel). Finally, we tested *eat-16; egl-10* double mutants and found that they showed a sensitivity to exogenous DA that was indistinguishable from *rsbp-1; egl-10* double mutants (Figure 5B, bottom panel). All together, our data indicates that RSBP-1 acts with EAT-16 and not with EGL-10 to modulate DA signaling in *C. elegans*.

### RSBP-1 and EAT-16 act downstream of the D1/DOP-1 receptor

In our previous analysis of DA signaling, we provided evidence suggesting that the R7 RGS protein EGL-10 inhibits DOP-3 signaling and that EAT-16 inhibits DOP-1 signaling [25]. In the present work, our data thus far suggest that: 1) RSBP-1 does not modulate D2-like DOP-3 signaling but rather might modulate D1-like DOP-1 signaling; and 2) RSBP-1 acts with EAT-16 and not EGL-10 to modulate DA signaling. Therefore, we next wanted to test more directly whether RSBP-1 acted downstream of the DOP-1 or the DOP-3 receptor.

**Figure 6:**
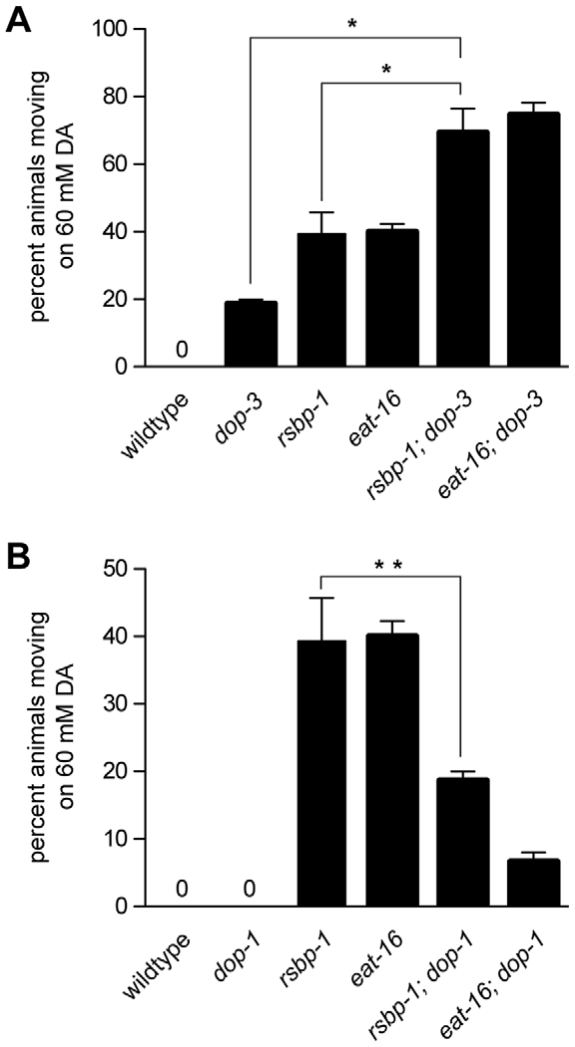
Quantitative analysis of dopamine response in *rsbp-1; dop-3* and *rsbp-1; dop-1* double mutants. Shown are the percentages of animals moving 20 min after being placed on agar plates containing 60 mM dopamine. Each data point represents the mean ± standard error of the mean (s.e.m.) for three trials totaling at least 75 animals. One-way ANOVA with Bonferroni post hoc test was used to compare all strains. Single asterisk indicates p<0.001, double asterisk indicates p<0.05. RSBP-1 acts with the R7 RGS protein EAT-16 to modulate signaling by the D1/DOP-1 receptor.

Since R7BP and R7 RGS proteins act together to inhibit receptor signaling in mammals (by accelerating the GTPase activity of Gα subunits), we predicted that mutations in R7BP would have opposite effects on behavior compared to mutations in the receptor whose signaling it normally inhibits. We have shown that both *rsbp-1* and *dop-3* mutants show similar defects in three separate DA-specific behaviors. Mutations in both genes cause resistance to exogenous DA (Figure 2A), suppress the locomotion defects of *dat-1* mutants (Figure 1), and cause similar defects in an animal's ability to slow in response to food (Figure 2B). These results indicate that RSBP-1 does not inhibit DOP-3 signaling. However, the data is also consistent with RSBP-1 and DOP-3 acting together to mediate DA signaling with RSBP-1 being required for DOP-3 function. If RSBP-1 is required for DOP-3 signaling, *dop-3* and *rsbp-1* mutants would be expected to show similar resistance to exogenous DA and *rsbp-1; dop-3* double mutants would be no more resistant to exogenous DA than either *dop-3* or *rsbp-1* single mutants. We found however, that *rsbp-1; dop-3* double mutants were significantly more resistant to exogenous DA than either *dop-3* or *rsbp-1* single mutants (*dop-3* single mutants 18% moving ±1%; *rsbp-1* single mutants 39%±6%; *rsbp-1; dop-3* double mutants 69% moving ±7%) (Figure 6A). Hence our data indicate that RSBP-1 is not required for DOP-3 signaling.

Since resistance to exogenous dopamine can be caused by either decreased DOP-3 signaling or increased DOP-1 signaling [25], [27], the DA resistance of *rsbp-1* mutants could be due to increased DOP-1 signaling. If RSBP-1 inhibits DOP-1 signaling we would expect *rsbp-1* and *dop-1* mutants to have opposite effects on DA sensitivity. Furthermore, if the DA resistance of *rsbp-1* mutants was due to increased DOP-1 signaling, we would expect that the DA resistance of *rsbp-1* mutants would be attenuated in *rsbp-1*; *dop-1* double mutant animals. Indeed, this is exactly what we observed. Unlike *rsbp-1* mutants, *dop-1* mutants are completely paralyzed by a 20 minute exposure to 60 mM exogenous DA (Figure 6B). At earlier time points and at lower concentrations of DA, we found that *dop-1* mutants were indeed more sensitive to exogenous DA than wild-type animals and thus the effect of the *dop-1* mutation is opposite that of the *rsbp-1* mutation (data not shown). Finally, we found that the DA resistance of *rsbp-1* mutants was attenuated when the DOP-1 receptor was removed. Whereas 39%±6% of *rsbp-1* mutants were resistant to 60 mM DA only 19%±1% of *rsbp-1; dop-1* double mutants were resistant.

All together, our results indicate that RSBP-1 acts selectively with the R7 RGS protein EAT-16 (and not the other R7 RGS protein; EGL-10) to modulate DA signaling. Further, we have shown that while EAT-16 and RSBP-1 are expressed together with both D1- and D2-like receptors (DOP-1 and DOP-3) in cholinergic cells they are able to selectively inhibit DOP-1 signaling in these cells.

## Discussion

### dsRNAi screen identified six genes required for DA signaling

In this study we performed a large-scale RNAi screen to identify genes that mediate endogenous DA signaling in *C. elegans*. This screen resulted in the identification of six genes, *unc-43*, *flp-1*, *grk-1*, *cat-1, eat-16, and rsbp-1* that were required for the execution of DA-mediated behaviors. *unc-43* encodes the homolog of calcium/calmodulin-dependent protein kinase (CamKII). Mammalian CamKII has been previously implicated in signaling by both D1-like [18], [51] and D2-like receptors [52] and thus could also play a role in DA signaling in *C. elegans*. *flp-1* encodes a family of eight FMRFamide-related invertebrate-specific peptides and may act upstream of both Gαo and Gαq signaling in *C. elegans* [53]. The identification of FLP-1 in our screen suggests that neuropeptide and DA signaling may act together to modulate locomotion behavior in *C. elegans*. *grk-1* encodes the worm homolog of the GRK4 family of G protein receptor kinases which include GRK4, 5, and 6. Members of this GRK family can regulate the activity of the D1 and D2 receptors [54], [55] and GRK6 knockout mice are hypersensitive to the stimulatory effects of cocaine and amphetamine likely through enhanced D2 receptor signaling [56], suggesting that D2 receptors may be a physiological target for phosphorylation by GRK6. It has not yet been determined whether *C. elegans* DA receptors are also regulated by GRKs, and future characterization of *grk-1* mutants may shed light on this process. Finally, we identified *cat-1* in our screen. *cat-1* encodes the monoamine vesicle transporter required to load dopamine and serotonin into synaptic vesicles [31]. The identification of CAT-1 in the screen validated our approach in two ways. First it indicated that the dsRNAi feeding approach was capable of knocking down gene expression in neurons, and second, it demonstrated that the screen could identify genes required for endogenous DA signaling.

In addition to these genes we also identified *eat-16* and *rsbp-1* in our screen and showed that they are required to modulate DOP-1 receptor signaling. EAT-16 is one of two R7 RGS proteins found in *C. elegans*, similar in both amino acid sequence and domain structure to the mammalian R7 RGS proteins RGS6, 7, 9, and 11, and RSBP-1 is homologous to the R7BP protein that is required for the stability and function of RGS9-2. Mutations in *eat-16* and *rsbp-1* cause similar DA-specific behavioral defects suggesting that these two proteins function together to mediate DA signaling in *C. elegans*.

### Dopamine signaling mechanisms appear to be conserved between mammals and *C. elegans*

We have now conducted two separate genetic screens to identify molecules that either mediate the response of animals to exogenous DA [25] or that modulate a DA-dependent locomotion behavior (this study). From these screens we have identified 14 proteins required for DA signaling in *C. elegans*. Of these, 13 of them (all except FLP-1) are homologous to mammalian proteins expressed in the brain and have been linked to DA signaling in mammals.

The similarities in DA signaling between *C. elegans* and mammals are evident both at the amino acid and the functional levels. For example, like in mammals, dopamine acts through D1- (DOP-1) and D2-like (DOP-3) receptors in *C. elegans* and these receptors are coupled to Gαq and Gαo subunits, [11], [16], [25], [57]. In *C. elegans* dopamine signaling is modulated by R7 RGS proteins that act as GAPs for these G protein subunits and there is building evidence that D2 receptor and Gαi/o signaling in the brain and in cell lines is modulated by R7 RGS proteins [41]–[43], [58], [59]. While no direct evidence yet indicates that D1 signaling may be modulated by R7 RGS proteins there is substantial evidence that Gαq signaling can be regulated by both RGS7 and RGS9 in mammalian cells [60]–[62]. Finally, DOP-1 and DOP-3 receptors act antagonistically in *C. elegans* to modulate acetylcholine release from motor neurons [27] and D1 and D2 receptors in mammals also have opposite effects on the release of both acetylcholine and GABA in the brain [63], [64].

In this study we have identified four new DA signaling genes, three of which are conserved in mammals (*grk-1*, *unc-43,* and *rsbp-1*). As mentioned earlier, GRK4–6 (homologs of GRK-1) and CamKII (homolog of UNC-43) have previously been implicated in DA signaling extending the similarity of DA signaling between the two species. While a role for R7BP (homolog of RSBP-1) in DA signaling has been recently described [35], it appears to act to inhibit D2-like signaling. This is not consistent with the role we have identified for RSBP-1 in *C. elegans* and suggests that perhaps this mechanism of signaling may not be conserved (see below).

### RSBP-1 and EAT-16 modulate DOP-1 (D1) receptor signaling in vivo

We have demonstrated that RSBP-1 acts with EAT-16 to inhibit signaling by the D1-like receptor DOP-1 and they do not modulate the activity of the D2-like receptor DOP-3. Several lines of evidence support this conclusion. First, mutations in *rsbp-1*, *eat-16*, and *dop-3* all blocked the ability of DA to inhibit swimming behavior (Figure 1) [26], [27]. Because RGS proteins act to inhibit G protein-coupled receptor signaling, it is predicted that mutations in *eat-16* and *rsbp-1* should cause behavioral effects opposite those caused by mutations in the receptor whose signaling they regulate. Second, mutations in *rsbp-1*, *eat-16*, and *dop-3* all caused defects in basal slowing, a second locomotory behavior controlled by endogenous DA signaling (Figure 2B and [25]). This also indicated that RSBP-1 and EAT-16 do not modulate DOP-3 signaling. In previous studies we showed that DOP-1 signaling had effects on these locomotion behaviors that were opposite to DOP-3 and thus the behavioral effects that we observe for *eat-16* and *rsbp-1* mutants are consistent with them acting as negative regulators of DOP-1 signaling. Third, while DOP-3 is expressed and functions in both GABAergic and cholinergic neurons to modulate locomotion behavior and response to DA, we found no function for RSBP-1 in the control of DA response in GABA cells (Figure 4). Thus, RSBP-1 likely does not modulate DOP-3 (D2) signaling in GABA motor neurons. We did observe a function for RSBP-1 when we expressed it in the cholinergic motor neurons where both DOP-1 and DOP-3 are expressed. This again suggested to us that RSBP-1 and EAT-16 might inhibit DOP-1 signaling instead of DOP-3 signaling.

We reasoned that the receptor signaling pathway normally inhibited by RSBP-1 and EAT-16 would be more active in *rsbp-1* and *eat-16* mutants and that this increased receptor signaling is what caused the altered response of *rsbp-1* and *eat-16* mutants to exogenous DA (Figure 2). To identify that receptor we combined mutations in *eat-16* and *rsbp-1* with mutations in *dop-1* and *dop-3* and found that the sensitivity of *rsbp-1* and *eat-16* mutants to DA was restored only when the DOP-1 receptor was removed demonstrating that the DOP-1 receptor is normally inhibited by EAT-16 and RSBP-1 (Figure 6). In contrast, the ability of *rsbp-1* and *eat-16* mutants to move in the presence of exogenous DA was increased further when the DOP-3 receptor was removed (Figure 6). Together, these data clearly demonstrate that EAT-16 and RSBP-1 act together to regulate DOP-1 and not DOP-3 signaling.

While we believe the mechanisms of DA signaling are conserved between *C. elegans* and mammals, these last results conflict with those from studies of RGS9-2 and R7BP function which suggest that these proteins modulate signaling by D2 receptors and not D1 receptors. [41]–[43]. For example, RGS9-2 colocalizes with D2 receptors and not D1 receptors in CHO cells [43]. Rats in which RGS9-2 was overexpressed unilaterally in the nucleus accumbens exhibited a turning behavior indicative of an imbalance in DA signaling when treated with a D2-selective agonist but not a D1-selective agonist [41]. Finally, the RGS domain of RGS9-2 blunted the effects of D2-selective agonists on calcium channel currents in dissociated striatal interneurons [42].

How can these apparently disparate results be reconciled? First, it is possible that R7 RGS proteins and R7BP target different G protein α subunits (and thus different receptors) depending upon the cell type in which they are expressed or perhaps even where in the cell they are found. For example, in addition to its apparent ability to inhibit D2 signaling, mammalian RGS9-2 can also inhibit NMDA and µ-opioid receptors in behaving animals and in biochemical assays in striatal extracts [65]–[67]. RGS9-2 can also modulate M2 muscarininic receptor activity when expressed in oocytes and in transfected CHO cells [68], [69]. In a similar way, we suspect that RSBP-1 and EAT-16 modulate signaling from other G protein-coupled receptors in addition to DOP-1 in *C. elegans*. For example, while EAT-16 and RSBP-1 are expressed in most or all neurons (Figure 3, [32], [33]) DOP-1 shows a more limited expression pattern [25]. Furthermore, RSBP-1 is expressed in both cholinergic and GABA motor neurons but DOP-1 is not expressed in GABA neurons. We also found functional evidence to suggest that RSBP-1 and EAT-16 regulate other G protein-coupled receptors. The DA resistance of *rsbp-1* and *eat-16* mutants can not be accounted for by unregulated activity of DOP-1 alone as *rsbp-1; dop-1* and *eat-16; dop-1* double mutants are still resistant to exogenous DA while *dop-1* single mutants are not (Figure 6). Thus it is possible that RGS9-2 (EAT-16) / R7BP (RSBP-1) complexes can modulate signaling by many receptors including both D1 and D2-like DA receptors. In further support of this, mammalian RGS9-2 is expressed in most or all neurons of the striatum including D2-expressing cells, D1- expressing cells, and cholinergic interneurons [41], [42].

The discrepancy might also be explained by our ability to examine the function of these signaling proteins *in vivo* in single cell types. In this work we directed the expression of transgenes using well-defined promoters that are active in very select cell types within the nervous system. Expressing these cell-specific transgenes in null mutant animals allowed us to test protein function and the interaction between two or more signaling proteins in single cell types in live, behaving animals. Mammalian promoters are more complex than those found in *C. elegans* and the number of well-characterized mammalian promoters is small. In their study of RGS9-2 function, Rahman et al. [41] drove expression of RGS9-2 in the striatum using viral-mediated overexpression techniques that do not permit cell-type selectivity but instead cause expression in most or all cells near the site of virus injection. As the striatum consists of a heterogeneous population of cell types, this approach will cause the increased expression of RGS9-2 in multiple cell types. Because RGS9-2 likely acts at many different types of G protein-coupled receptors, changes in its expression which are not restricted in the cell-type affected can have confounding effects on the activities of many receptors, cells, and circuits that ultimately control a behavior. It is possible, therefore, that such overexpression strategies could cause complex signaling effects that confound experimental results and obscure the true function (or site of action) of the gene under investigation. Whether or not RGS9-2 and R7BP act together to modulate D1 receptor signaling in the brain might be tested by using the D1 receptor promoter to drive the expression of these proteins (or RNAi against them) only in D1 receptor expressing neurons [70].

## Materials and Methods

### Nematode Culture

Worm strains were maintained at 20°C under standard conditions and double and triple mutants were generated using standard genetic methods [71]. The wild-type strain used was Bristol N2. Strains analyzed in this study were: LX1270: *rsbp-1(vs163) I,* DA702: *eat-16(ad702) I,* XP139: *dat-1(ok157) III* (4x outcrossed), NM1378: *unc-43(js125) IV,* XP461: *flp-1(ok2811) IV* (4x outcrossed), VC10127: *grk-1*(*gk1192*) (not outcrossed), MT8504: *egl-10(md176) V,* LX645: *dop-1(vs100) X,* and LX703: *dop-3(vs106) X.* CB1112: *cat-2 (e1112) III*, XP154: *eat-16(ad702); dat-1(ok157)*, XP453: *rsbp-1(vs163); dat-1(ok157),* XP405: *dat-1(ok157); unc-43(js125),* XP464: *dat-1(ok157); flp-1(ok2811),* XP465: *dat-1(ok157); grk-1(gk1192),* XP390: *dat-1(ok157); egl-10(md176),* XP391: *rsbp-1(vs163); egl-10(md176),* XP349: *eat-16(ad702) rsbp-1(vs163),* XP392: *rsbp-1(vs163); dop-3(vs106),* XP389: *eat-16(ad702); dop-3(vs106),* XP357: *rsbp-1(vs163); dop-1(vs100),* XP388: *eat-16(ad702), dop-1(vs100),* LX1313: *eat-16(ad702); egl-10(md176),* XP462: *eat-16(ad702) rsbp-1(vs163); dat-1(ok157),* XP463: *rsbp-1(vs163), dat-1(ok157); egl-10(md176)*. The RNAi screen was conducted using XP292: *dat-1(ok157); eri-1(mg366*); *lin-15b(n744)*.

### SWIP suppressor screen

The triple mutant strain XP292 was used for the *dat-1* SWIP suppressor screen. A *C. elegans* feeding dsRNA library was purchased from Open Biosystems. The efficacy of dsRNA knockdown was strictly dependent on maintenance of dsRNA plasmid in the bacteria used to feed animals. We found that plasmid was lost at high frequency from bacteria grown in ampicillin up to 2 mg/ml. We therefore did not use this antibiotic to grow bacteria. Bacteria grown in liquid media were less prone to plasmid loss than bacteria grown on solid agar surfaces and we found that liquid cultures containing 500 µg/ml carbenicillin was sufficient to ensure 100%±15% plasmid retention, while 2 mg/ml carbenicillin was needed to ensure plasmid retention when bacteria were grown on agar plates.

The 96-well plates containing dsRNA-expressing bacterial clones were thawed and replica-plated into 150 µl LB medium containing 8% glycerol, 16 µg/ml tetracycline, 3.2 mg/ml carbenicillin using a 96-pin Boekel replicator and were grown for 8–10 hr at 37°C with shaking (450 RPM, orbit 3 mm) to generate duplicate library plates. This unusually high concentration of carbenicillin was necessary as ∼50% of the bacteria in each culture from the original library plates did not contain plasmid. Bacteria from duplicate library plates were transferred using a 96-pin replicator to agar-filled omniplates containing LB media with 16µg/ml tetracycline and 2 mg/ml carbenicillin. The bacteria on these omniplates were grown at 37°C for 15–18 hr and were then stored inverted at 4°C for up to one week. Bacteria from these omniplates served as the source of food for the screen. Library bacterial clones were replicated from omniplates into deep well (2 ml) 96-well dishes each well containing 1 ml LB media with 16 µg/ml tetracycline and 500 µg/ml carbenicillin. These deep well dishes were shaken flat for 24 hr at 37°C at 650 RPM (orbit 3 mm) until the cultures were saturated. After shaking, 150µl of each culture was transferred to the surface of NGM agar media containing 2 mg/ml carbenicillin, 1mM IPTG but without tetracycline in 12 well plates and the bacteria were allowed to absorb into the agar for 24 hr at room temperature. The next day, 30±10 synchronized L1 XP292 animals were placed in each well of the 12-well NGM plates and stored at 20°C for 5–6 days in a humidified chamber. For each 96-well library plate tested we included one test well that contained bacteria with empty vector (pL4440) as control. After 5–6 days, when the L1 animals had fully developed, laid progeny, and the oldest progeny were L4 stage, the entire population of animals in each well was washed off with water and transferred immediately to empty 12-well plates and were tested for swimming-induced paralysis (SWIP) suppression after 10 min. The wells in which >40% of tested animals showed sustained swimming after 10 min were considered to be positive “hits” and were retested in triplicate for SWIP using the exact same conditions as in the original screen. Only dsRNAs that retested at least twice were selected for further study.

### Behavioral assays

SWIP assays on individual strains were performed by picking 10 L4 animals away from food and then placing them in a 50 µL water droplet on a Menzel Glaser 10-well diagnostic slide (model X1XER308B#) and scoring for swimming after 10 min. Swimming was defined as the presence of free alternating body bends characteristic of *C. elegans* swimming behavior [72]. This assay was repeated for a total of 50 animals per strain.

DA dose-response assays were performed as described previously [25]. Briefly ∼25 young adults for each strain were incubated undisturbed for 20 min on plates containing the indicated concentration of DA, and then scored for paralysis. Animals were considered paralyzed if they did not exhibit at least one spontaneous body bend in a 20 sec observation period. Assays were repeated in triplicate for a total of at least 75 animals per strain.

Basal slowing assays were done as previously described [25]. Briefly, the locomotion rates of staged young adult animals were quantified by counting the number of body bends completed in five consecutive 20 sec intervals in the presence or absence of HB101 bacteria. Plates with bacteria were prepared by spreading 100 µl of HB101 bacteria (A_600_ = 0.70–0.75) across each plate and incubation overnight at 37°C. Data were collected for six animals per strain per condition (food, no food) for a total of 30 measurements per condition. Percent slowing was calculated by dividing the difference between locomotion rates on and off food by the locomotion rate off food.

### Transgenic animals

To examine the expression pattern of RSBP-1 we constructed a transgene with the promoter of *rsbp-1* (3,260 nucleotide basepairs upstream of the start codon) fused to the coding sequence for GFP (pCL114) and injected it, together with a *lin-15*-rescuing plasmid as marker (pL15EK, both plasmids injected at 50 ng/μl), into MT8189 *(lin-15(n765))* animals to generate the strain XP369. Transgenic animals were identified by the absence of the multiple vulva phenotype typical of *lin-15(n765)* mutants and by green fluorescence. To generate double transgenic lines containing both *rsbp-1p*::GFP and *dop-3p*::RFP transgenes, we crossed XP369 animals and LX811 (*lin-15B(n745) X; vsIs33*, [*dop-3p::*RFP] animals. For double transgenic lines between *rsbp-1p*::GFP and *unc-47p*::mCherry, we crossed XP369 and XP300 (*unc-47p::mcherry* transgenic line, generous gift of M. Francis). For generating double transgenic lines between *dop-1p*::GFP and *rsbp-1p*::mCherry, a *rsbp-1p::*mCherry construct (pCL133, promoter identical to pCL114) (at 50 ng/μl) was injected into *dop-1p*::GFP animals (LX798) and the double transgenic animals were identified by their green and red fluorescence.

### Cell-specific rescue

For generating rescue transgenic lines each rescue plasmid [*rsbp-1p:: rsbp-1* (pCL127, containing 3,260 bp of *rsbp-1* promoter region fused to cDNA encoding RSBP-1), *unc-119p::rsbp-1* (pCL129, containing 2,181 bp of *unc-119* promoter region fused to cDNA encoding RSBP-1*), unc-17p::rsbp-1* (pCL130 containing 3,249 bp of *unc-17* promoter region fused to cDNA encoding RSBP-1), *unc-47p::rsbp-1* (pCL131, containing 257 bp of *unc-47* promoter region fused to cDNA encoding RSBP-1) *and myo-3p::rsbp-1* (pCL132, containing 2,379 bp except 7 bp immediately upstream of the start codon of *myo-3* promoter region fused to cDNA encoding RSBP-1)], was injected at 50 ng/μl along with the co-injection marker, *myo-2p*::GFP (pJK4) at 30 ng/μl into *rsbp-1* mutant animals (LX1270). For control lines, empty vectors containing only the respective promoters were injected at 50 ng/μl along with *myo-2p*::GFP at 30 ng/μl. For both rescue and control plasmids, 2–3 independent lines were generated and tested side by side for the rescue of the dopamine sensitivity of *rsbp-1* mutants. For each line 25 animals in triplicate were tested on exogenous dopamine for a total of 75 animals per line.

### Statistical analysis

Comparisons shown in Figures 1, 2B, 4, 5A, and 6 were done using one-way ANOVA with Bonferroni post hoc test. In Figures 2A and 5B we compared the curves of each mutant to the wildtype or other appropriate control (see result section) using a two-way ANOVA with repeated measures followed by a Bonferroni multiple comparisons post hoc test.
